# Glycated albumin in pregnancy: reference intervals establishment and its predictive value in adverse pregnancy outcomes

**DOI:** 10.1186/s12884-019-2704-x

**Published:** 2020-01-03

**Authors:** Ying Dong, Yanhong Zhai, Jing Wang, Yi Chen, Xin Xie, Chunhong Zhang, Jingrui Liu, Yifan Lu, Guodong Tang, Lican Han, Lin Li, Zheng Cao

**Affiliations:** 1 Department of Laboratory Medicine, Beijing Obstetrics and Gynecology Hospital, Capital Medical University, 251 Yaojiayuan Road, Beijing, 100026 China; 2 Obstetrical Department, Beijing Obstetrics and Gynecology Hospital, Capital Medical University, Beijing, China; 3 Prenatal Diagnosis Center, Beijing Haidian Maternal and Child Health Hospital, Beijing, China; 4 Central Laboratory, Beijing Obstetrics and Gynecology Hospital, Capital Medical University, Beijing, China

**Keywords:** Glycated albumin, Gestational diabetes mellitus, Adverse pregnancy outcomes, Birth weight

## Abstract

**Background:**

Many efforts have been focused on the alternative glycemic marker glycated albumin (GlyA) and its application in pregnancy during which profound physiological changes take place. Our objective was to determine the reference intervals (RIs) of GlyA in healthy Chinese pregnant women and to assess the predictive value of serum GlyA in adverse pregnancy outcomes.

**Methods:**

Totally 421 healthy subjects including 137 in the first trimester, 152 in the second trimester, and 132 in the third trimester were enrolled from March to July 2019, for the purpose of establishing the trimester-specific RIs of GlyA. In addition, 67 pregnant women diagnosed with GDM were enrolled at 24–28 weeks of gestation. The diagnostic value of GlyA for GDM patients was evaluated and compared with that of fasting plasma glucose (FPG) at 24–28 weeks of gestation. The association between GlyA in the late pregnancy and the adverse pregnancy outcomes was analyzed with the data collected from January to June 2018 at our hospital.

**Results:**

The estimated RIs of GlyA in present study were 11.26–15.10%, 10.04–13.50%, and 9.76–13.09% in the first, second, and third trimesters respectively. The areas under receiver operating characteristic (ROC) curves were 0.503 for GlyA and 0.705 for FPG. More importantly, the GlyA level in the third trimester was not more elevated in the patients with adverse pregnancy outcomes including large for gestational age (LGA), preterm delivery, hypertension and preeclampsia (PE). The exception was made with the GDM patients who suffered from postpartum hemorrhage and had significantly higher GlyA levels than the control group.

**Conclusions:**

Our results showed that the GlyA was continuously decreased as the gestational age went up. The GlyA testing has limited value in diagnosing GDM and predicting adverse pregnancy outcomes.

## Background

Gestational diabetes mellitus (GDM) is defined as diabetes diagnosed in the second or third trimester of pregnancy that was not clearly overt diabetes before pregnancy [1]. As one of the most common pregnant complications, the prevalence of GDM has been increased during the last decades and is estimated to continue to increase in the future [2]. According to a research based on over 125 million pregnant subjects between 1979 and 2010, the increased prevalence of GDM can be mainly attributed to high maternal age and body mass index (BMI) [3]. Adverse pregnancy outcomes of GDM affect both mothers and newborns in short and long terms. The women who were diagnosed with GDM have higher risk of type 2 diabetes and cardiovascular diseases after delivery [4]. In addition, GDM is closely associated with metabolism disorders, hypertensive disorders, preeclampsia (PE), large for gestational age (LGA, birth weight above the 90th percentile for gestational age), cesarean delivery and related birth injury in perinatal period [3, 4].

Strict glycemic control is the key to prevent or decrease adverse perinatal complications. With a half-life of 8–12 weeks for red blood cells, the glycated hemoglobin A1c (HbA1c) can reflect ambient blood glucose level in the past 2–3 months [5]. It has been widely used in monitoring glycemic state and guiding clinical therapy for diabetes patients. The HbA1c threshold of 6.5% for diabetes mellitus diagnosis is supported by the DETECT-2 collaboration [6]. However, the glycemic status may not be accurately monitored by the HbA1c level in some situations such as hemolytic anemia, iron deficiency anemia, uremia, hemoglobinopathies and pregnancy [7]. In normal pregnancy, the HbA1c level presents biphasic changes, including a significant decrease in the second trimester and a slowly elevation to its peak level in the third trimester [8]. For example, Richard et al. found that the HbA1c concentration reached the nadir level at the 24 week’s gestation [9]. Alternatively, glycated albumin (GlyA) is formed through a nonenzymatic reaction between blood glucose and serum albumin. As not affected by hemoglobin metabolism or iron-deficient anemia, GlyA is often recommended for clinical practice on glycemic control in pregnancy where HbA1c is not appropriate to implement [7, 10].

Only a few articles focused on establishing the GlyA reference ranges in pregnancy. In a multi-center study based on the Japanese population, the GlyA level was significantly decreased in the second and the third trimesters compared with the first trimester [8]. In another study with 1479 normal pregnant women, the mean levels of GlyA were 11.53% in the 24–28 weeks and 10.23% in the 36–38 weeks [11]. According to the above studies in which the GlyA level was significantly decreased during pregnancy, it is important to establish trimester-specific reference intervals (RIs). Moreover, it is still controversial if higher GlyA level is associated with maternal and complications in GDM patients [12, 13]. The purpose of this study was to determine the RIs of GlyA in Chinese pregnant women, and to assess the predictive value of GlyA in adverse pregnancy outcomes.

## Methods

### Study population

For the GlyA RIs establishment, a cross-section study was performed. Any patient that had already been recruited was not enrolled for the second time during the entire sample collection period. Totally 605 singleton pregnant women attending routine check-ups in the Beijing Obstetrics and Gynecology Hospital from March to July 2019 were initially recruited. The following exclusion criteria were applied in our study: pre-pregnancy BMI ≥ 25 kg/m^2^ [14], liver dysfunction with elevated transaminases, abnormal kidney function with elevated Cr and Bun, serum albumin lower than 32 g/L, elevated fasting plasma glucose (FPG) (normal range of 3.9–6.1 mmol/L in the first trimester) or GDM, lipid metabolism disorders, clinical or subclinical thyroid dysfunction. Eventually, 421 out of 605 healthy subjects were enrolled for RIs establishment, including 137 in the first trimester, 152 in the second trimester, and 132 in the third trimester. A sample size of more than 120 was used to derive the RI by the nonparametric approach according to the Clinical and Laboratory Standards Institute (CLSI) guideline EP28-A3C [15]. The demographic characteristics of healthy subjects in three trimesters were shown in Table 1.

**Table 1:** Characteristics of healthy pregnant women in three trimesters

	First trimester	Second trimester	Third trimester
Number	137	152	132
Age (years)	30.84 ± 3.19	31.04 ± 3.37	30.94 ± 4.76
Pre-pregnancy BMI (kg/m^2^)	21.01 ± 2.19	20.28 ± 1.97	20.87 ± 1.95
Gestational age (weeks)	6.81 ± 1.11	23.30 ± 2.44	31.79 ± 0.83

*BMI* body mass index

In present study, all the GDM patents were diagnosed by the 75-g oral glucose tolerance test (OGTT) according to the International Association of Diabetes and Pregnancy study Groups (IADPSG) 2010 criteria [16]). Specifically, GDM was determined by meeting one of the three following criteria: FPG ≥5.1 mmol/L, 1-h postprandial blood glucose ≥10.0 mmol/L, or 2-h postprandial blood glucose ≥8.5 mmol/L. There were 67 pregnant women diagnosed with GDM and enrolled at 24–28 weeks of gestation. The collected serum samples of the recruited healthy and GDM subjects were stored at − 80 °C before the GlyA testing.

**Fig. 1:**
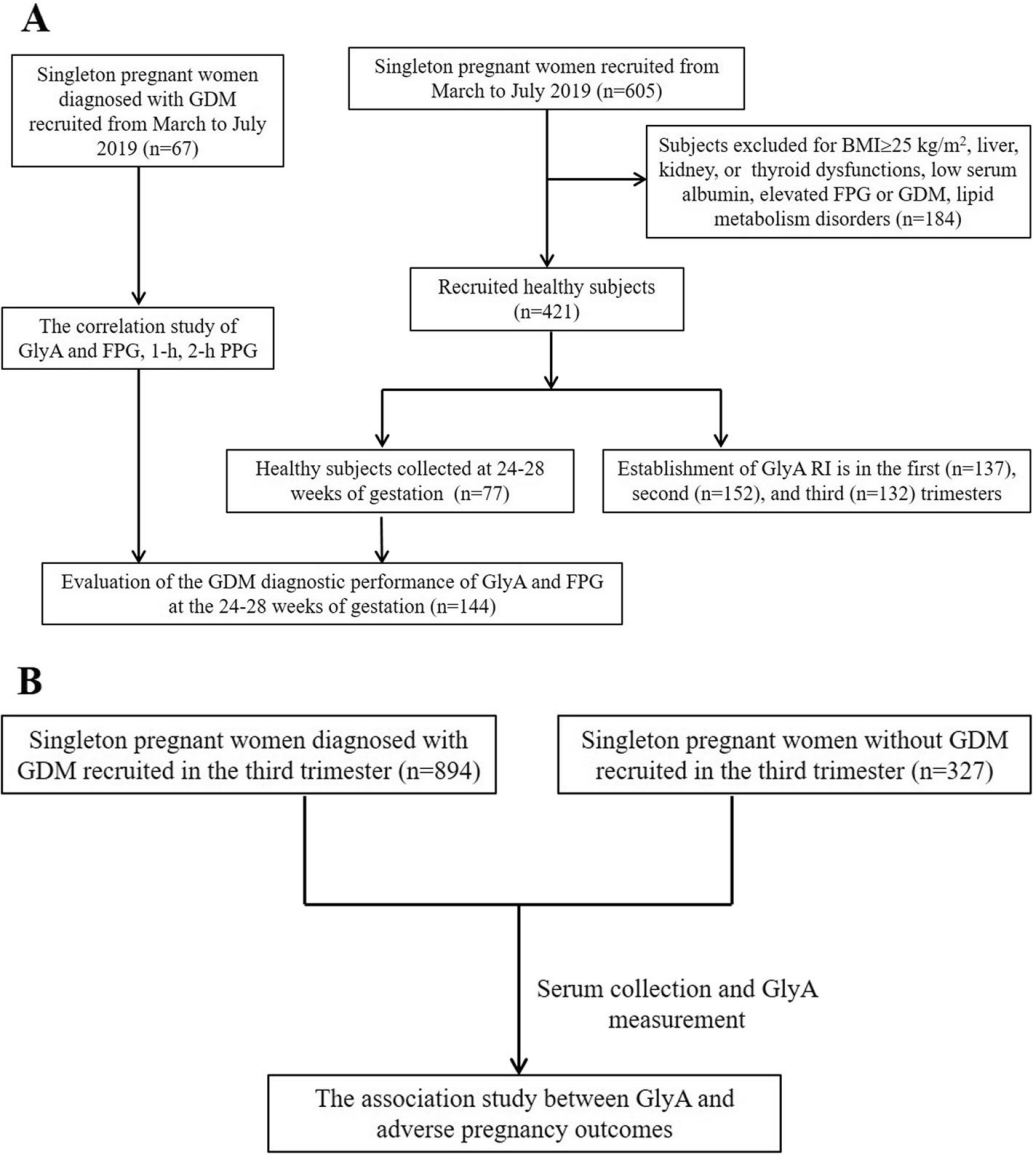
Schematic diagram for patient recruitment and study design. **a** the study design for GlyA reference intervals (RIs) establishment and its evaluation in GDM diagnosis; **b** the study design for the associations between third trimester GlyA and adverse pregnancy outcomes. FPG, fasting plasma glucose; PPG, postprandial plasma glucose

In addition, 894 pregnant subjects that were diagnosed with GDM between January and June 2018 and delivered live newborns at our hospital were analyzed in the association study with the GlyA values of the third trimester and adverse pregnant outcomes, including preterm delivery, postpartum hemorrhage, PE, hypertension and LGA. Meanwhile, 327 pregnant women without GDM or pre-pregnant DM were recruited in the third trimester as non-GDM group, with their sera samples tested for GlyA and pregnancy outcomes recorded after delivery. A schematic diagram was made for illustrating the flow of participants and study design (Fig. 1).

### GlyA measurement

The GlyA results were calculated as the ratio of glycated albumin over albumin. Specifically, the glycated albumin and albumin were assayed on the fully automated biochemical analyzer Abbott C16000 (Abbott Park, IL, USA) using the peroxidase method (Glycated albumin assay kit, Catalog Number 3R43.09, Beijing Strong Biotechnologies Inc., Beijing, China). Total albumin was quantified by the bromocresol purple method (Glycated albumin assay kit, Catalog Number 3R43.10, Beijing Strong Biotechnologies Inc., Beijing, China).

### Statistical analysis

The Dixon method was applied to remove the outliers from the dataset in the RI study. The numerical data of GlyA level was presented as the mean ± standard deviation (SD) if it was normally distributed or was expressed as median followed with interquartile range if the data was skewed. The RIs were estimated by the IBM SPSS Statistic 21 (SPSS Inc., Chicago, IL, USA, RRID:SCR_002865) using the nonparametric approach. To examine the statistical significance of GlyA levels between any two groups, the Student’s t test was used and *P* < 0.05 was considered to be statistically significant. The correlation between GlyA and FPG, 1 h-, 2 h- postprandial plasma glucose was determined separately by the Pearson’s correlation coefficient test. The chi-square test was performed to compare the incidence rates of greater-than-upper-limit for GlyA levels in the third trimester between the groups with and without adverse pregnancy outcomes. The odds ratio (OR) was applied to evaluate the association between the adverse outcomes and the elevation of GlyA. The receiver operating characteristic (ROC) curve analysis was performed to compare the diagnostic power between GlyA and fasting plasma glucose at the 24–28 weeks of gestation for GDM and assess the predictive value of GlyA in adverse pregnant outcomes in women with and without GDM.

## Results

The ages of subjects, the pre-pregnancy BMI and the weeks of gestation were summarized in Table 1. There was no significant difference for maternal ages and pre-pregnancy BMI when compared between different trimesters.

With normal distributions examined by the Kolmogorov-Smirnov test (data not shown), the means of the GlyA level were 13.22 ± 0.98% in the first trimester (less than 13 weeks), 11.89 ± 0.88% in the second trimester (14–27 weeks), and 11.33 ± 0.86% in the third trimester (28–40 weeks). According to the CLSI guideline EP28-A3C (original GlyA data available in the Additional file 1: Table S1), the 2.5th and 97.5th were used as the lower and upper limits of RIs respectively. The 90% confidence interval (CI) of the lower and upper limits was calculated with bootstrap method. As shown in Table 2, the RIs of GlyA were 11.26–15.10%, 10.06–13.51%, and 9.76–13.09% in the first, second and third trimesters respectively.

**Table 2:** Trimester-specific reference intervals of glycated albumin

	First trimester	Second trimester	Third trimester
GlyA(%), mean ± SD	13.22 ± 0.98	11.89 ± 0.88	11.33 ± 0.86
2.5% percentile (90% CI)	11.26 (10.88, 11.77)	10.06 (9.57, 10.43)	9.76 (9.37, 9.99)
97.5% percentile (90% CI)	15.10 (14.68, 16.30)	13.51 (13.26, 13.85)	13.09 (12.69,13.43)

*GlyA* glycated albumin, *SD* standard deviation, *CI* confidence interval

**Fig. 2:**
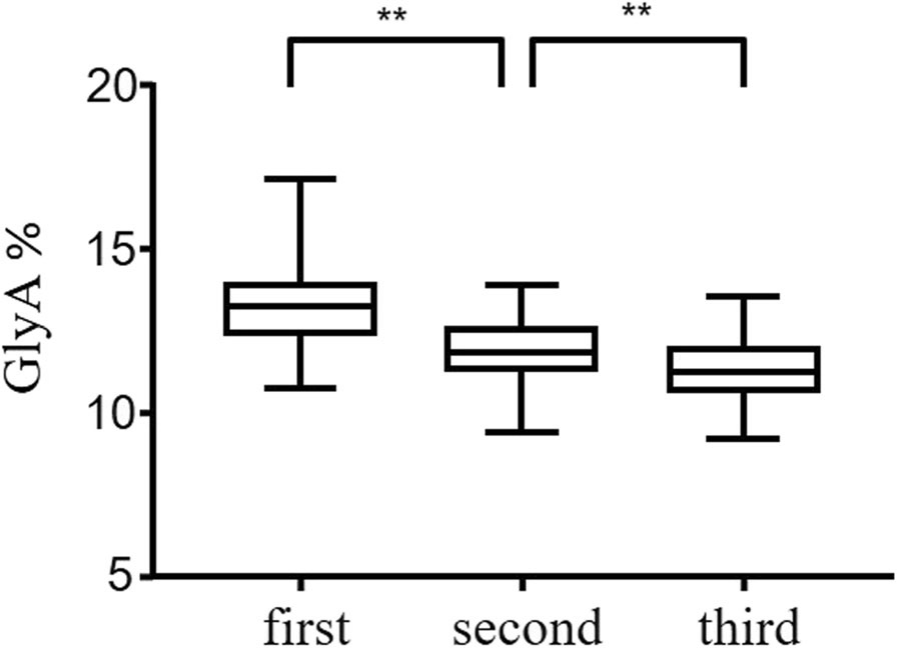
Box plots of glycated albumin in the healthy pregnant women recruited in present study. *indicates *P* < 0.05; **indicates *P* < 0.001. Student’s t test was performed to calculate the *P* value

As shown in Fig. 2, the GlyA level was significant lower in the second trimester than that in the first trimester (*P* < 0.001). This decreasing tendency was also recognized when comparing the GlyA in the second with that in the third trimesters (*P* < 0.001).

**Fig. 3:**
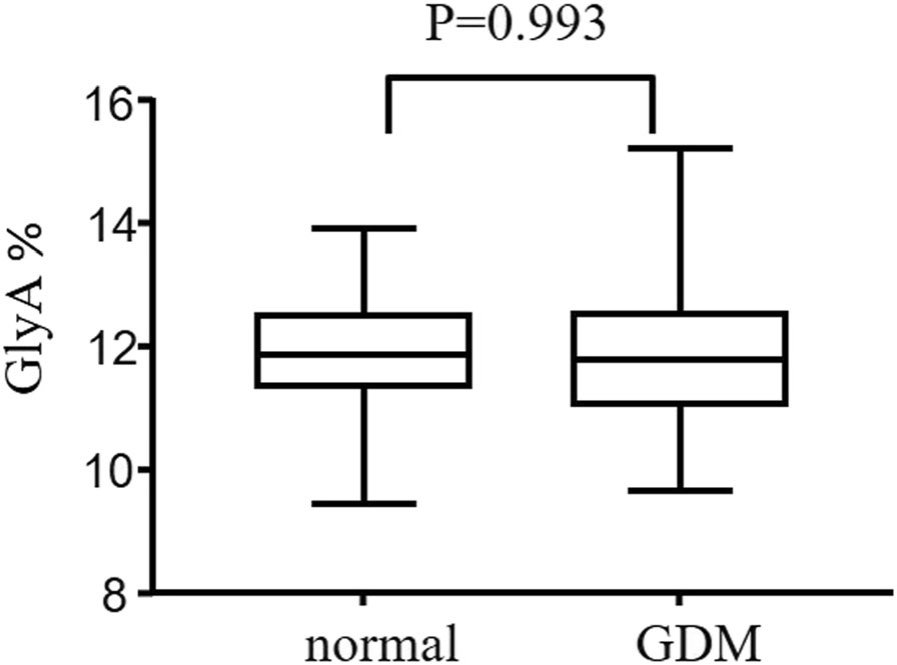
Box plots of glycated albumin in women with and without GDM at the 24–28 weeks of gestation. Student’s t test was performed to calculate the *P* value

**Fig. 4:**
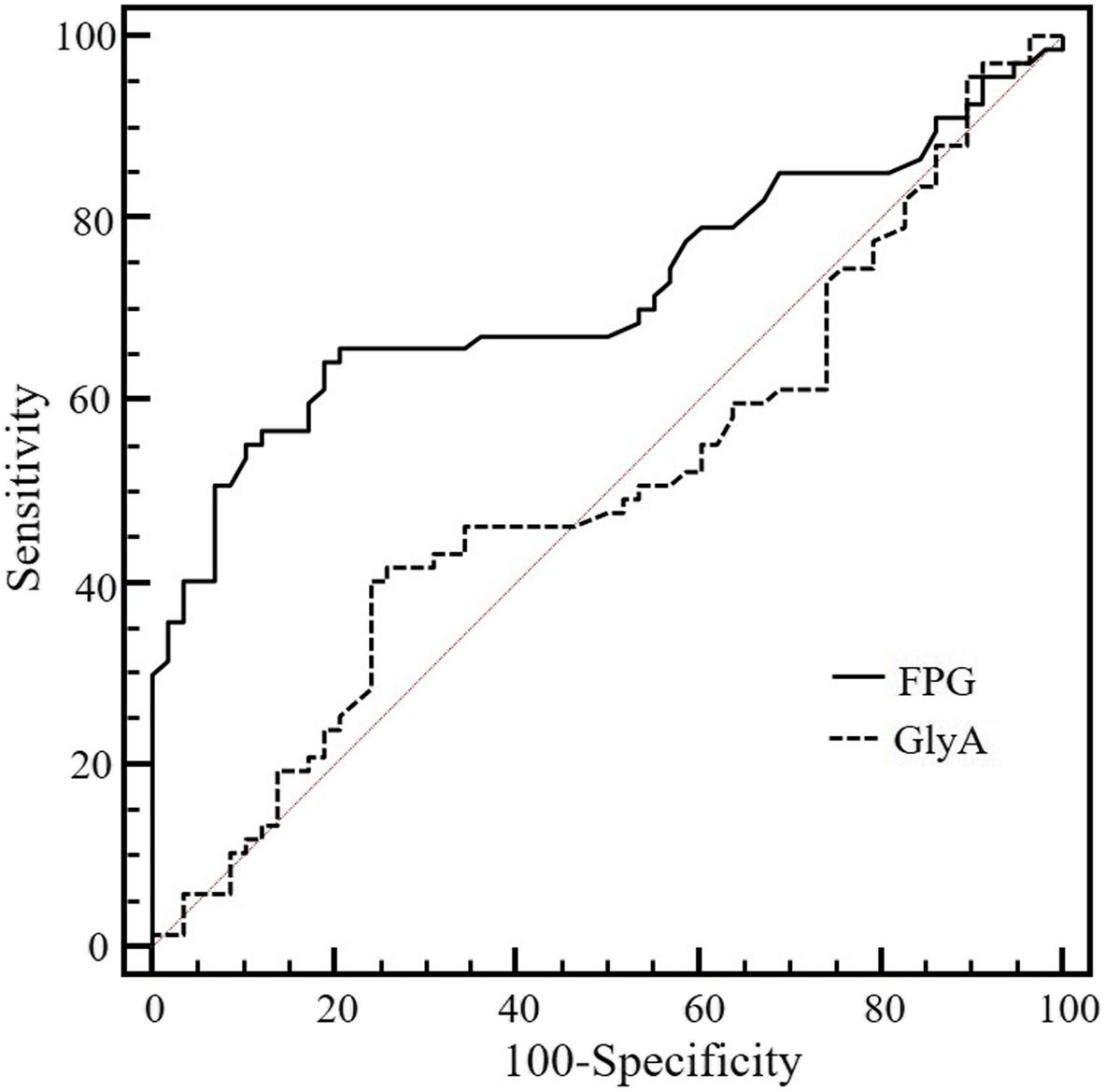
Receiver operating characteristic (ROC) curve analysis of GlyA and FPG at the 24–28 weeks of gestation for diagnosing GDM. The areas under ROC curve were 0.503, (*P* = 0.957) for GlyA and 0.705 (*P* < 0.001) for FPG

Interestingly, there was no statistical difference of the GlyA levels between the GDM group and the healthy pregnancy at 24–28 weeks of gestation (Fig. 3) (Additional file 1: Table S2). When compared with the GlyA, the FPG had a better discriminating power for GDM, with the areas under curve (AUC) of 0.705 (*P* < 0.001) in the ROC analysis (Fig. 4). Furthermore, the GlyA level did not show a linear correlation with the FPG (R^2^ = 0.021; *P* = 0.245), 1-h (R^2^ = 0.002; *P* = 0.711) or 2-h postprandial plasma glucose (R^2^ = 0.013; *P* = 0.349) in women with GDM at 24–28 weeks of gestation.

**Fig. 5:**
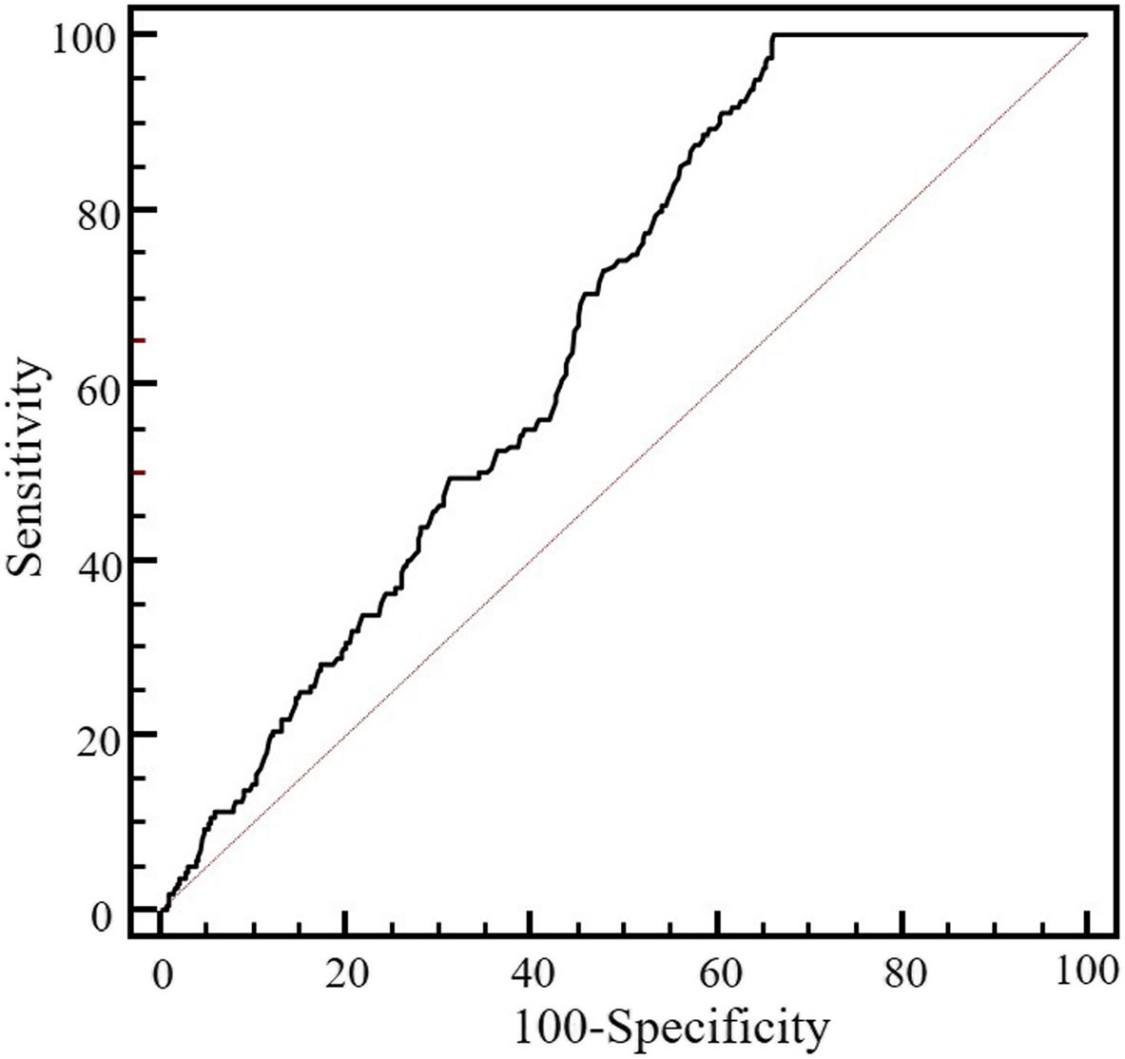
Receiver operating characteristic (ROC) curve analysis of GlyA for predicting postpartum hemorrhage in women with GDM

In the association study with the GDM patients, the third trimester GlyA level did not exhibit a higher incidence rate of greater-than-upper-limit (Gly A > 13.09%) in the patients with preterm delivery, hypertension, PE or LGA, when compared with the control group (Table 3). The exception was a made in the patients with postpartum hemorrhage, in which higher GlyA level was observed (*P* = 0.03, OR = 1.57, Table 3). In addition, the GlyA showed better discriminating power by ROC curves (AUC = 0.663) (Fig. 5) in the patients with postpartum hemorrhage than in the patients with the rest adverse outcomes (ROC data not shown). By contrast, in the analyses with the non-GDM patients, the GlyA was not statistically different in the patients with or without adverse pregnancy outcomes.

**Table 3:** Comparisons of glycated albumin of pregnant women with and without adverse pregnancy outcomes

	Case N/total	Control N/total	Odds ratio	*P*-value
Non-GDM group (*n* = 327)				
Preterm delivery	0/5	4/322	0.00	1.00
Postpartum hemorrhage	0/14	4/313	0.00	1.00
Preeclampsia	0/16	4/311	0.00	1.00
Hypertension	0/69	4/258	0.00	0.58
LGA	0/12	4/310	0.00	1.00
GDM group (*n* = 894)				
Preterm delivery	13/63	212/831	0.76	0.40
Postpartum hemorrhage	37/160	118/734	1.57	0.03
Preeclampsia	10/60	215/834	0.58	0.12
Hypertension	9/45	180/849	0.93	0.85
LGA	31/114	181/717	1.11	0.66

case: patients with the specific adverse pregnancy outcome; control: patients without specific adverse pregnancy outcome. LGA: large for gestation age. N: the number of subjects with GlyA greater-than-upper-limit (13.09%). total: the total number of the subjects with (case) or without (control) specific adverse pregnancy outcome. Chi-square test was performed for *P* value calculation. For the LGA study, the subjects with preterm delivery were excluded

## Discussion

The GlyA is considered a more sensitive blood glucose indicator than the HbA1c in pregnancy with a shorter half-life of around 2–3 weeks [7, 17]. Kouzuma et al. [18] reported that the GlyA reference interval in the non-pregnant Americans with normal glucose tolerance was 11.9–15.8%. However, with the observation made in present study, the GlyA level was gradually decreased when the gestation age went up. This physiological change of serum GlyA level during pregnancy has been reported both in healthy pregnant women and in those diagnosed with GDM [11]. Hiramatsu et al. [8] showed that the normal ranges of GlyA in healthy Japanese women were 12.2–16.6% in first trimester, 11.8–15.6% in second trimester and 11.3–15.5% in third trimester with both lower and upper limits higher than those reported in our study, suggesting the lack of the GlyA reagent uniformity and the potential impact of different ethnic background. Even with the same GlyA reagent (Lucica GlyA-L, Asahi Kasei, Tokyo) that was used by the above Japanese research group, the mean GlyA value of the 24–28 weeks gestations with Chinese pregnant women was still lower than that of the Japanese pregnant women [11].

Both BMI and urinary protein has been reported as two important factors influencing GlyA levels during pregnancy. The GlyA concentrations were found much lower in the high BMI group (≥25 kg/m^2^) than in the low BMI group (18.5–25 kg/m^2^); the GlyA was also lower in the pregnant women with elevated urinary protein [8]. Selvin et al. [19] also reported an inverse association between GlyA and BMI. However, the underlying mechanism for decreased GlyA level in the subjects with higher BMI still remains unknown. One hypothesis was that the chronic inflammation related turnover of negative acute-phase proteins might have led to the decrease of GlyA [20]. Glomerular filtration rate (GFR) could also be attributed to the changes of GlyA level during pregnancy. It has been reported that the GlyA level was inversely increased when the estimated GFR (eGFR) was decreased due to renal dysfunction in both diabetic and nondiabetic population [21]. As pregnancy progressed, physiologically elevated eGFR may also result in the decrease of GlyA.

We compared the GlyA levels of pregnant women with and without GDM at the 24–28 weeks of gestation and found no significant difference (*P* = 0.993) between the two groups. This result was consistent with a study conducted by Zhu et al. [22], in which no statistical GlyA difference was observed between the patients with and without GDM. In current practice, GDM was diagnosed during 24–28 weeks of gestations by FPG and postprandial plasma glucose levels. As the HbA1c concentration was influenced by the half-life of red blood cell which could be prolonged by iron-deficiency during pregnancy, its application in GDM diagnosis has not been widely recommended [23, 24]. As a non-traditional glycemic marker, the limited diagnostic value of GlyA in GDM has been reported in several articles. Zhu et al. [22] reported that the AUC values were 0.726 for FPG and only 0.542 for GlyA in the second trimester. Similar observation was made by Saglam et al. [25], with the AUC of GlyA being 0.550 in the GDM diagnosis. In our study, we also found a higher diagnostic value of FPG (AUC = 0.721) than that of GlyA (AUC = 0.509) during the 24–28 weeks of gestation. Besides, there was no linear correlation between the GlyA and the FPG, 1-h or 2-h postprandial plasma glucose (PPG) with the collected serum samples. However, Koga et al. [26] reported that GlyA could accurately reflect the postprandial plasma glucose. Huang et al. [27] showed that the FPG and the GlyA values exhibited a significant correlation in all pregnant women although the linear coefficient was only 0.103. In a non-pregnant population including subjects with diabetes mellitus, impaired glucose regulation and normal glucose regulation, the linear correlation between GlyA and FPG had been observed with a better coefficient factor (R^2^ = 0.41) and GlyA was also observed statistically correlated with 2-h PPG (R2 = 0.43) [28]. Interestingly, a study with the euglycaemia group showed that the GlyA levels did not correlate with PPG and was likely to reflect incidental glycation throughout the albumin lifespan [29]. Plus, as a distinct clinical entity, GDM is at least partially introduced by the profound physiological changes during pregnancy, such as hormones or mediators secreted by placenta. And in some sense, GDM could be seen as the early stage of type-2 diabetes mellitus [30]. Therefore, together with the insufficient GDM discrimination power in our study, we hypothesize that the GlyA formation in GDM patients may behave more like that in euglycemic women and could not well predict FPG or PPG response.

Fetal macrosomia which is defined as infant birth weight above a specific threshold, historically 4000 g or 4500 g, is a common adverse neonatal outcome of GDM. With the threshold of 4000 g, the incident rate of macrosomia is 15–45% of women diagnosed with GDM compared with 12% of normal women [31]. The Pedersen’s hypothesis explaining the pathophysiology of macrosomia is that the maternal hyperglycemia leads to fetal hyperglycemia and hyperinsulinemia which further result in protein and fat stores in fetus [31]. However, due to lack of standard diagnosis criteria for macrosomia, LGA has been alternatively used to assess excessive fetal growth based on the population birthweight curves. Here we explored the association between GlyA and birth weight to evaluate the third trimester GlyA in predicting LGA. Our results showed that the GlyA was not significantly different (with GlyA = 13.09% as threshold, Table 3) in the patients with or without LGA in both GDM and non-GDM groups (Table 2). Interestingly, Zhang et al. [32] conducted a study involving 242 Chinese pregnant women with GDM and they found the GlyA level had no association with neonate birth weight in the late pregnancy. Another study with Chinese women diagnosed with GDM showed that the GlyA level at 36–38 weeks of gestation had no difference between the maternal group with birth weight of 3000–3499 g and the group with birth weight of 3500–3999 g [11]. In a multicenter study including 136 Japanese diabetic pregnant women, the incidence of large-for-date showed no statistical difference between the group of GlyA ≥15.8% group and the group of GlyA < 15.8% group with *P* = 0.071 [13]. However, the above negative findings about the GlyA prediction on birth weight have been controversial. In a study of 42 Japanese women with GDM, the maternal GlyA level was significantly higher in the group of infants with large-for-date status [33]. It has been also reported that an average increase of 76.1 g in birth weight was observed per 1% maternal GlyA elevation [10]. According to the work by Catalano et al. [34], although no significant difference of the infant birth weight between the GDM and the non-GDM groups was found, the fat mass of infants was changed in the same direction as the maternal blood glucose level. Therefore, the blood glucose or GlyA may have a better predictive value for neonatal body compositions (such as fat mass) than simple body weight.

Unfortunately, to our best knowledge, the previous publication that was directly focused the association between GlyA level in late pregnancy and maternal adverse outcomes was limited. In a recent study by Kumari et al. with Indian population, the prevalence of postpartum hemorrhage in GDM and non-GDM groups was not significantly different [35]. Interestingly, a Chinese group reported that poor glycemic control monitored by FPG in the GDM patients had led to elevated postpartum hemorrhage [36]. Similarly, our data also showed that in the GDM group with postpartum hemorrhage more patients had elevated GlyA, suggesting the importance of glycemic control in late pregnancy. It has been relatively well established that GDM posed an increased risk of hypertension in patients of different ethnic backgrounds including Chinese population [37–39]. In contrast, whether the rate of preterm delivery increases in pregnancies complicated with GDM remained debatable [40]. Nevertheless, elevated GlyA did not seem to aggravate the prevalence of hypertension or preterm delivery in the patients with or without GDM (Table 3).

Although it was not statistically different, lower incidence rate of great-than-upper-limit for the GlyA level was observed with the GDM patients who suffered from preeclampsia (10/60 = 16.7% in the PE case group vs 215/834 = 25.8% in the control group, OR = 0.58) (Table 3). In the study focused on the relationship between the serum albumin and the preeclampsia, it was found that women in the preeclampsia group displayed significantly lower level of serum albumin than those in the normal group. It was proposed that albumin might function in suppressing vascular oxidative stress and preventing endothelial dysfunction [41]. Therefore, the decreased albumin and the resulting decreased GlyA may have made the patients more vulnerable to preeclampsia. In another relevant research, Wang et al. reported that glycated serum protein (measured as serum fructosamine) was decreased in PE patient during pregnancy [42].

## Conclusions

The trimester-specific RIs of GlyA showed an obvious decreasing trend throughout the entire pregnancy. As a short-term glycemic control indicator, the GlyA level has limited value in diagnosing GDM at the 24–28 weeks of gestation and in predicting adverse pregnancy outcomes.

## Supplementary information

**Additional file 1.** The original glycated albumin and glucose measurements in the trimester reference intervals establishment and the GDM diagnosis study. **Table S1.** GlyA data of healthy pregnant women in three trimesters. **Table S2.** GlyA and FPG data of women with and without GDM at the 24-28 weeks of gestation.

## Data Availability

The original GlyA and FPG datasets generated during the current study are available and provided as Additional file 1: Table S1 and Table S2. Alternatively, the Additional file are available in the Open Science Framework Repository (www.osf.io, DOI 10.17605/OSF.IO/AN6R4). However, according the patients’ verbal consents, their biometrics and pregnancy outcomes are only available from the corresponding author on reasonable request.

## Abbreviations

AUC: Areas under ROC curve
BMI: Body mass index
CI: Confidence interval
CLSI: Clinical and Laboratory Standards Institute
FPG: Fasting plasma glucose
GDM: Gestational diabetes mellitus
GFR: Glomerular filtration rate
GlyA: Glycated albumin
HbA1c: Glycated hemoglobin A1c
LGA: Large for gestational age
OGTT: Oral glucose tolerance test
PE: Preeclampsia
RI: Reference interval
ROC: Receiver operating characteristic
